# Romanian Dentists’ Perceptions on Molar Incisor Hypomineralization—A Questionnaire-Based Study

**DOI:** 10.3390/children12060680

**Published:** 2025-05-25

**Authors:** Beatrice Ciocan, Lucian Cristian Petcu, Rodica Luca

**Affiliations:** 1 Doctoral School, Faculty of Dentistry, Carol Davila University of Medicine and Pharmacy, 37 Dionisie Lupu, 020021 Bucharest, Romania; 2 Department of Biophysics, Biostatistics, and Medical Informatics, Faculty of Dentistry, Ovidius University of Constanța, 1 Aleea Universității, 900470 Constanta, Romania; 3 Department of Pedodontics, Faculty of Dentistry, Carol Davila University of Medicine and Pharmacy, 37 Dionisie Lupu, 020021 Bucharest, Romania

**Keywords:** MIH awareness, MIH perceptions, MIH Romania, dental hypomineralization, pedodontics

## Abstract

Molar Incisor Hypomineralization (MIH) is a common dental condition that affects the mineralization of the enamel, primarily affecting the first permanent molars and often the incisors. This condition can lead to a wide range of clinical presentations, from mild opacities to severe post-eruptive breakdown, which can significantly impact a child’s oral health and quality of life. **Background/Objectives**: The prevalence and complex management of MIH have posed a significant challenge for dental practitioners. Our preceding investigation found that 14.3% of school-aged children have MIH. Based on this finding, we wanted to understand what other Romanian dental professionals think about this condition. Therefore, the aim of the present study was to assess the awareness, perception, and clinical management approaches of Romanian dentists toward MIH in order to inform future educational strategies and contribute to the development of dedicated preventive programs. **Methods**: To gain a comprehensive understanding of MIH in actual clinical settings, we developed and administered a questionnaire consisting of three distinct sections. Our objective was to capture the collective knowledge and perspectives of dental practitioners. We distributed the survey, which included 14 pertinent questions, to a large professional group of Romanian dentists. **Results**: This study collected responses from 219 Romanian dental practitioners (median age: 34 years) about their experiences with MIH. The vast majority (86.76%) had encountered MIH cases in their practice, with half reporting moderate prevalence among their patients. The most frequently observed complications were hypersensitivity (41.95%), pulp exposure (33.33%), and failed restorations (24.71%). While adhesive restorations were identified as the overall preferred treatment approach (70.00%), notable differences emerged in both clinical complications encountered and therapeutic approaches implemented across dental specialties. There was near-unanimous agreement on the importance of early MIH diagnosis (99.09%), and almost all participants (98.63%) expressed a desire for more information about this condition, demonstrating high awareness and concern about MIH among Romanian dental professionals. **Conclusions**: This study highlights that general dentists, endodontists, and pedodontists encounter MIH patients frequently in their practice, emphasizing the critical need to enhance awareness and education about MIH among both dental professionals and the general public.

## 1. Introduction

Molar Incisor Hypomineralization (MIH) is defined as a qualitative enamel developmental defect of systemic origin that affects one or more first permanent molars with or without the involvement of permanent incisors [1].

The term MIH was described by Weerheijm et al. in 2001 and was adopted by the international dental community following the European Academy of Paediatric Dentistry (EAPD) meeting held in Athens in 2003, addressing the issue of hypomineralized incisors and molars [1].

Systemic hypomineralization can affect both permanent dentition, referred to as MIH [1], and primary dentition, specifically second primary molars, which is described in the literature as either DMH (Deciduous Molar Hypomineralization) [2] or HSPMs (Hypomineralized Second Primary Molars) [3]. Although HSPMs (DMH) are a distinct clinical entity from MIH, they are considered a predictive factor for the future development of MIH [4].

Despite the lack of consensus regarding this fact, a plausible explanation for the concurrent presentation is that the development of second primary molars begins approximately simultaneously with the development of the first permanent molars and permanent incisors. However, the maturation of permanent teeth progresses more slowly, and if a disruptive factor intervenes during the period when these teeth are developing concurrently, hypomineralization manifestations will be observed in both dentitions [5].

The hypomineralization defects observed in MIH cases exhibit a spectrum of characteristics, ranging from mild opacities to severe post-eruptive enamel breakdown and, in some instances, even total tooth loss that may affect from one to four first permanent molars [4].

MIH develops through complex, interconnected pathways rather than a single cause. Research has identified several potential MIH risk factors, including premature birth, oxygen deprivation during the perinatal period, and various childhood health conditions. Among these associated illnesses are infections of the urinary and respiratory systems, asthma, and gastrointestinal disorders, though this represents only a partial list of potential contributing factors [6].

Recent global research demonstrates that MIH affects between 9.4% [7] and 13.1% [8] of children worldwide, with significant regional variation observed across continents.

The latest comprehensive analysis of 80 studies, including over 191,000 children, revealed the Americas exhibit the highest prevalence of MIH (17.7%), followed by Asia (10.7%), Europe (7.3%), and Africa (4.9%), which has the lowest MIH prevalence, highlighting the importance of geographic and potential socioeconomic factors in the epidemiology and management of this dental condition [7].

Dental complications associated with MIH include hypersensitive teeth, rapid caries progression, impaired mastication due to rapid tooth structure loss, and finally, but equally important, esthetic repercussions. These issues can affect patients’ quality of life and represent a challenge for dentists. MIH clearly warrants increased attention as a global dental public health concern [9].

Given the notable prevalence of MIH uncovered in our prior research among Romanian school children [10], it is imperative to investigate the knowledge and perspectives of Romanian dental professionals on this condition.

Therefore, the aim of this study was to explore the awareness and perception of MIH among Romanian dentists.

## 2. Materials and Methods

We conducted a cross-sectional study targeting Romanian dental professionals, utilizing a comprehensive distribution strategy to ensure broad reach.

The questionnaire was distributed via the following multiple complementary channels to maximize its reach across diverse dental practice contexts: (1) in-person distribution at two national professional conferences and one international professional conference, where it was specifically distributed to Romanian conationals, with all the in-person distribution facilitated via a QR code; (2) postings in the oldest dedicated professional social media group with a combined membership of approximately 14,207 Romanian dental practitioners; and (3) distribution through university resident coordinators at three dental teaching institutions in Romania who encouraged participation among faculty and affiliated practitioners. This multi-channel approach was designed to reduce potential selection bias by reaching dentists with varying professional engagement patterns.

The study population comprised general dentists and other dental specialists who voluntarily completed the survey.

The inclusion criteria specified licensed dentists actively practicing in either the public health system or the private sector in Romania. While not formally stated as exclusion criteria in the original protocol, the distribution channels naturally excluded dental students, retired practitioners, and non-practicing dental professionals, as the questionnaire was explicitly presented as being for currently practicing clinicians.

We employed a voluntary response sampling strategy, inviting all eligible dental practitioners to participate during the designated data collection period. A total of 219 respondents completed the questionnaire, offering sufficient variability across age, gender, and dental specialties to address the study objectives related to MIH awareness, perception, and management. The final sample size is comparable to, or larger than, those reported in similar cross-sectional studies in the field.

Participation in this study was entirely voluntary, and all participants provided informed consent before completing the questionnaire. At the beginning of the online form (administered via Google Forms, LLC, Alphabet Inc., Mountain View, CA, USA, in 2023), participants were presented with an introductory statement explaining the purpose of this study, the voluntary nature of participation, and the confidentiality of their responses. The statement informed respondents that the data would be used exclusively for research purposes and provided the researcher’s contact information for any further questions. By proceeding with the questionnaire, participants acknowledged that they understood the conditions and consented to participate anonymously.

Data collection spanned a nine-month period, using a modified questionnaire adapted from previous validated studies [11,12,13,14,15].

The modifications we implemented to our questionnaire, compared to the previous studies, were methodologically driven to enhance both validity and completion rates. We eliminated clinical case photographs to prevent potential bias in initial treatment perceptions and to reduce the survey completion time.

In contrast to similar instruments containing four to seven sections, we consolidated our questionnaire into three principal sections, strategically merging general MIH knowledge with therapeutic approaches into a single cohesive section to maintain continuity and improve response fluidity.

Our distribution methodology differed from the aforementioned questionnaire-based studies through a combined approach that integrated distribution channels described across multiple previous studies. We employed both physical distribution methods (via QR code scanning) and digital dissemination through university networks and social media platforms, creating a more comprehensive reach across the professional dental community.

Prior to the distribution of the questionnaire, a preliminary test was conducted with a small group of practicing clinicians in order to ensure its functionality and clarity, as well as to verify compliance with the estimated completion time. This pilot testing contributed to refining the structure and content of the questionnaire and confirmed its appropriateness for the target population.

The questionnaire was structured to have three distinct sections. The first section gathered participants’ background variables including age, gender, qualifications, and practice characteristics. The second section assessed variables related to their understanding of MIH’s prevalence, clinical features, complications, and management approaches. The third section explored variables concerning their perceptions and educational interests regarding this dental condition. Thus, the variables that were collected for each study participant included demographic information, knowledge and awareness of MIH, and attitudes toward continuing education on this topic.

The ethical approval of the study protocol and the informed consent form were provided by the Research Ethics Committee of the Carol Davila University of Medicine and Pharmacy, Bucharest, Romania (approval number: 3365/2025).

Descriptive statistical analysis was performed in IBM SPSS Statistics version 25 (IBM Corp., Armonk, NY, USA).

## 3. Results

The total number of respondents was 219, who answered the 14 questions of the questionnaire structured into three sections.

In Section 2 of the questionnaire—Awareness and Perception of MIH—respondents who had not encountered MIH in their practice discontinued answering the questionnaire (*n* = 29).

### 3.1. Demographics

The study sample consisted of 219 respondents, whose ages ranged from 24 to 59 years. The mean age was 35.01 years, with a standard deviation of 6.51 years, indicating a moderately varied age distribution. The median age was 34 years, while the interquartile range extended from 30 to 38 years, reflecting a relatively concentrated middle-aged group within the sample.

Among the 219 respondents, a significant majority of respondents were female, representing 85.39% of the total sample, while 14.61% were male. This gender distribution highlights a clear predominance of women within the surveyed population, which may reflect broader demographic trends within the dental profession or specific subspecialties included in this study.

Of the total respondents, the majority reported practicing general dentistry (53.42%), followed by endodontics (19.63%) and pediatric dentistry (11.42%). Smaller proportions were observed in periodontology (8.22%), orthodontics (3.65%), oral surgery (2.28%), and oral pathology (1.37%). This distribution reflects a participant pool predominantly composed of general practitioners, who typically serve as the primary providers for early diagnosis and intervention in conditions, such as MIH. The notable representation of pediatric dentists and endodontists further underscores the clinical relevance of MIH within these specialties, given the condition’s occurrence in children and its potential progression to pulpal involvement. Conversely, the limited representation of surgical and oral pathology specialists may suggest that advanced interventions or complex diagnostic cases are less frequently associated with routine MIH management (Table 1).

### 3.2. Awareness and Perception of MIH

The second part of the questionnaire assessed the dentists’ knowledge and awareness of MIH, which revealed the following key findings. A substantial majority of respondents (86.76%, *n* = 190) reported encountering clinical cases of MIH in their practice, indicating a high level of exposure to the condition. In this section, the respondents who had not encountered MIH in their practice discontinued answering the questionnaire (*n* = 29).

Among the respondents who had encountered MIH cases, the self-reported frequency of occurrence in their patient population was as follows:

- 26.84% of respondents reported encountering MIH frequently;
- 42.63% of respondents reported encountering MIH occasionally;
- 30.53% of respondents reported encountering MIH rarely.

When estimating the prevalence of MIH within their patient populations, 50.00% of dentists perceived it as moderate, 33.16% as high, and 16.84% as low (Table 2).

The most frequently observed clinical signs included white-yellow or brown enamel discolorations (81.58%), followed by post-eruptive enamel breakdown, atypical restorations, complicated caries, and cold sensitivity. Regarding complications, dentists most commonly reported hypersensitivity (41.95%), pulpal exposure (33.33%), and failed restorations (24.71%).

Upon closely examining the complications associated with MIH, a crosstab analysis revealed notable differences in the distribution of MIH-related complications encountered across specialties. Pedodontists most frequently reported dentin hypersensitivity (73.68%), while general practitioners and endodontists showed a more balanced distribution between pulp exposure and hypersensitivity. Orthodontists and periodontists reported the highest rates of restoration failures (57.14% and 58.33%, respectively). Surgeons and oral pathologists predominantly observed pulp exposure, with no reported cases of restoration failure. These trends highlight the influence of specialty-specific clinical focus on the perception and reporting of MIH-related complications (Table 3).

In terms of treatment strategies, restorative treatments were the preferred option for 70% of clinicians, with others opting for referral to a specialist, pulp therapy, or endodontic treatment (Table 4).

Following an in-depth analysis of treatment preferences among dental specialties, a crosstab analysis revealed that restorative treatments were the most commonly preferred option across specialties, particularly among general practitioners (77.36%) and pedodontists (72.00%), who identified them as the treatment of choice for MIH. Endodontists showed a more diversified therapeutic approach, with notable preferences for pulp therapy (18.75%) and endodontic treatment (25.00%), consistent with their clinical expertise. Orthodontists (42.86%) and oral surgeons (50.00%) were more likely to refer cases to other specialists, suggesting limited direct involvement in restorative management (Table 5).

Notably, 40.53% of the respondents who had managed MIH cases had encountered tooth extractions due to the severity of the condition.

The vast majority of respondents emphasized the significance of early diagnosis for the successful management of Molar Incisor Hypomineralization (99.09%).

### 3.3. Continuing Education and Training on MIH

**Figure 1:**
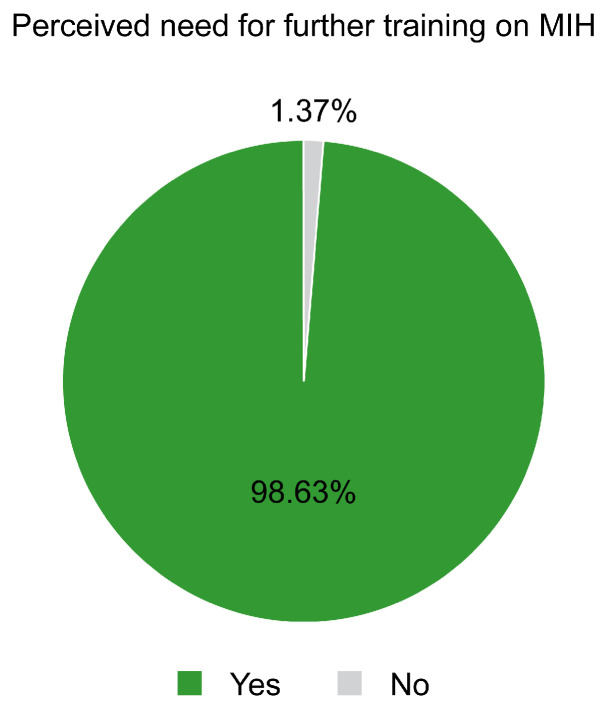
Perceived need for further training on MIH.

In terms of continuing professional education, only 35.16% of the respondents reported having previously attended courses or training sessions specifically focused on MIH, while the remaining 64.84% had not received any formal instruction on the topic. Despite this, a substantial 98.63% of respondents expressed the view that further training on MIH is necessary, as depicted in Figure 1, highlighting a widespread recognition of the need for enhanced education in this area. These observations underscore a significant gap between the current level of formal training and the clinical demands of managing MIH, suggesting that targeted continuing education programs could play a critical role in improving both diagnostic and therapeutic outcomes.

## 4. Discussion

The aim of this study was to describe the perceptions of Romanian dental practitioners regarding MIH in order to contribute to the development of dedicated preventive programs, considering the limited number of studies on this topic in Romania. A majority of respondents (86.76%) had encountered MIH in clinical practice, perceiving it as a condition of moderate prevalence. The most frequently reported complications included dentin hypersensitivity and pulp exposure, while restorative treatment was the most commonly preferred management option (70%). Despite limited prior training (35.16%), nearly all participants (98.63%) emphasized the need for further education on MIH.

This investigation is particularly timely and relevant considering the socio-educational and socioeconomic context in Romania. From an educational point of view, pediatric dentistry only achieved formal recognition as a specialization in 2016 [16] with the inaugural cohort of specialists completing their postgraduate training in 2019—a significant advancement in the Romanian dental education framework.

In the residency curriculum, developmental dental anomalies, including MIH, are discussed for 38 h [17]. In the undergraduate curriculum, the topic of MIH is addressed within the context of odontogenesis disturbances and developmental dental anomalies, totaling 4 h [18,19]. This aspect is particularly significant because, for the cohort of general dental practitioners, this may represent their only formal education regarding MIH.

From an economic perspective, Romanian national health insurance covers only basic dental services. Advanced dental interventions typically require families to pay significant costs themselves [20].

The widespread preference for restorative treatments across all dental specialties (70% overall), regardless of clinical severity or specialty focus, suggests a possible lack of tailored protocols and insufficient clinical decision-making tools for MIH management. Even specialists who might be expected to employ more diverse treatment strategies showed a strong preference for basic restorative approaches, with general practitioners (77.36%) and pediatric dentists (72.00%) particularly favoring this approach.

A concerning finding from our investigation was the substantial proportion of practitioners (40.53%) who reported encountering tooth extractions due to MIH. This notable extraction frequency may indicate systemic deficiencies in early detection or intervention protocols. Such findings underscore the need for improved early detection strategies and more effective interventions before cases progress to stages where extraction becomes the only viable option. In an attempt to study the percentage of molars extracted due to MIH, a study from the Department of Pediatric Dentistry at Carol Davila University of Medicine and Pharmacy in Bucharest [21] retrospectively analyzed 414 first permanent molars with MIH. They found that only 30% presented with mild forms, while 43.71% had moderate hypomineralization and 26.08% had severe forms. Dental caries affected 71.25% of MIH molars, with extraction indicated in 19.64% of cases and 9.17% actually extracted. The relatively high average age of the children included in this study likely contributed to the increased severity of MIH observed, suggesting that early intervention at ages 6–7, through school screening programs, could significantly reduce complications and the need for extractions through preventive and conservative approaches.

To the best of our knowledge, this study is one of the earliest systematic evaluations of Romanian dental professionals’ awareness, perceptions, and clinical experience related to MIH.

While various European studies have explored dentists’ knowledge and attitudes towards MIH, the available data are limited in the context of Romania, with only one study conducted on this topic [22].

Our study may contribute to the existing knowledge, offering new insights by covering a much larger, diverse sample across Romania, rather than being limited to a single county; by combining in-person, digital, and academic distribution networks to broaden its reach; and by employing crosstab analyses to compare complications and treatment approaches across dental specialties, thereby providing deeper and more nuanced clinical perspectives.

Differing from that study, our study shows a notably higher prevalence of dentists encountering MIH in their practice (86.76%) compared to the reference data, where only 70% of dental practitioners reported dealing with this syndrome. Our findings show a lower preference for restorative treatments (70%) compared to the reference study, where dental practitioners opted for direct reconstructions at a higher rate (83%), indicating a notable difference in treatment approach selection. We showed a greater need for education, with 98.63% of dentists desiring an improved understanding of MIH complications and 64.84% reporting not having received any formal MIH training, which aligns with the reference study, where 76% of dentists received no information on MIH and 81.3% wanted further information on the topic [22].

When comparing our findings to their European counterparts, the contextual factors previously mentioned help explain the observed differences. The similar frequency of MIH encounters between Romanian practitioners and those in Spain [11] and Greece [14], contrasted with higher regular encounters in Norway (92%) [12] and Ireland (89%) [13], likely reflects differences in surveillance systems rather than true prevalence variations.

Countries with longer-established pediatric specializations have developed more robust early detection protocols, explaining the higher reported encounter rates.

In terms of restorative preferences, our participants predominantly relied on basic restorative treatments and vital pulp therapies, with limited use of crowns—an approach that contrasts with the broader material diversity and more frequent use of preformed crowns observed in Greek [14] and Spanish [11] cohorts. This can be attributed to Romania’s dental insurance structure, which provides minimal coverage for advanced pediatric procedures, creating financial barriers to optimal care that do not exist to the same degree in countries with more comprehensive dental coverage [20].

Our findings align with a recent national analysis from Germany, highlighting the high MIH encounter rate among practitioners (86.8% of Romanian and 99.2% of German dentists reporting MIH encounters in practice), the significant perceived clinical relevance of MIH as a substantial challenge in daily practice (98.6% and 92.5%, respectively, perceiving MIH as a significant clinical problem), and the commonly reported clinical manifestations, particularly yellow-brown enamel discolorations (81.6% in our sample; 81% in theirs) as the primary clinical sign, which were consistently observed across both national samples [23].

Additionally, our study highlighted a substantial gap in training, as over 64% of respondents had never received formal education on MIH, and nearly all expressed the need for enhanced understanding—echoing similar educational deficiencies identified in Spain, Norway, and Greece, where continuing professional development on MIH remains a recognized and pressing need.

MIH is a widespread clinical issue [11,12,13,14]. Dentists report difficulty in diagnosing and managing MIH and consistently request more education on its causes, diagnosis, and treatment. The varied responses highlight the need for ongoing professional development in this area.

Compared to studies from India [24], Australia [25], Hong Kong [26], and Kuwait [15], our findings show that while MIH is commonly encountered by dental practitioners (86.76%), it is reported less frequently encountered than in India and Kuwait, where weekly encounters are more common.

Geographic variations in MIH encounter frequency likely reflect differences in practice patterns, patient demographics, and healthcare delivery frameworks that influence practitioner exposure to and documentation of hypomineralization defects.

Treatment in our sample relied mainly on basic restorations, with the limited use of crowns or preventive methods, in contrast to the broader use of preformed metal crowns observed in Australia, India, and Kuwait.

The preference for restorative treatments among our respondents (70%) underlines the importance of selecting materials with favorable mechanical and adhesive properties. As glass-ionomer cements are often recommended in MIH cases for their fluoride release and chemical bonding, research into their physical characteristics remains highly relevant [27].

The treatment approach differences reflect distinctive healthcare delivery systems and educational frameworks.

A significant training gap was evident, as 64.84% of our respondents had no MIH-specific education, mirroring global calls for improved training seen in over 85% of practitioners in the comparison studies.

In summary, our findings reveal that MIH constitutes a significant clinical challenge for Romanian dental professionals, with 86.76% encountering such cases despite only 35.16% having received formal training on the condition. The near-unanimous recognition (99.09% of respondents) of the importance of early MIH diagnosis reflects the growing professional awareness of MIH’s progressive nature.

The overwhelming consensus among practitioners (98.63%) regarding the need for additional education underscores a significant gap in professional development that must be addressed to improve MIH management outcomes.

These findings align with international recommendations, notably Fernández-Bonet (2023) [28], who strongly urges all European dental schools to give greater importance to MIH within their curricula, arguing that proper training can significantly improve young patients’ care and advance the collective understanding of the condition while also questioning whether similar gaps in MIH knowledge have been observed by other European professors.

This parallel between practitioner needs and academic recommendations highlights both the international trends and Romania-specific training and practice requirements that must be addressed to enhance MIH management outcomes.

This study provides insights with direct implications for both clinical practice and dental education. Romanian dentists frequently encounter MIH in their clinical work, yet most have not received formal training on the condition. These insights highlight the need to incorporate MIH-related content into undergraduate curricula and to create dedicated continuing education programs. Enhancing professional training in this area can support earlier diagnosis and promote consistent, evidence-based management strategies.

Similar to the findings by Laganà et al. (2025), who reported significant orthodontic treatment needs among school-aged children in Rome and emphasized the value of public health screening, our study highlights the need for dedicated preventive programs targeting MIH—especially given the high level of clinical encounters and the lack of formal training among Romanian dental practitioners [29].

Based on our empirical findings, we propose a multi-faceted approach to address this educational lacuna.

Firstly, the integration of MIH-specific educational modules into dental curricula at both undergraduate and postgraduate levels is warranted. Such educational frameworks should encompass etiopathogenesis, diagnostic methodologies, severity classification systems, and evidence-based management protocols stratified by clinical presentation. Particular emphasis should be placed on early detection paradigms to mitigate progression to severe manifestations necessitating extraction.

Secondly, given the preponderance of general dental practitioners in our respondent cohort (53.42%) and their pivotal role as primary diagnosticians, enhanced interdisciplinary collaboration frameworks between generalists and pediatric specialists are imperative. The establishment of formalized referral pathways and standardized management protocols could significantly augment early detection rates and optimize therapeutic outcomes.

Additionally, the implementation of standardized school-based screening programs represents a critical preventive strategy. Given that many respondents in our study may not have encountered MIH cases immediately after eruption, when the defects are categorized as mild defects—thus limiting early intervention opportunities—systematic screening initiatives in educational institutions would facilitate the identification of MIH-affected children at earlier stages when therapeutic interventions are maximally efficacious and minimally invasive. Such programs could integrate with existing school health frameworks and incorporate appropriate referral mechanisms to specialized care pathways.

Thirdly, the development of standardized clinical decision support instruments appears essential. Such tools—comprising diagnostic algorithms, severity assessment matrices, and treatment selection frameworks—would facilitate evidence-based intervention selection predicated on clinical presentation rather than defaulting to conventional restorative approaches irrespective of case complexity. Furthermore, in view of the overwhelming interest in continuing education (98.63% of respondents), the implementation of targeted professional development initiatives is indicated, with a particular focus on evidence-based management strategies that transcend basic restorative interventions for advanced cases. National clinical guidelines, coupled with enhanced surveillance mechanisms, would standardize therapeutic approaches and promote earlier intervention while being appropriately contextualized within the Romanian healthcare system’s financial parameters.

Finally, longitudinal research investigating treatment outcomes within the Romanian population would generate valuable evidence to inform clinical practice, particularly comparative analyses of cost effectiveness and clinical efficacy across various intervention modalities and severity gradients.

Our study comes with a set of limitations. The non-probabilistic sampling approach represents a significant methodological limitation of our investigation. Despite comprehensive efforts to disseminate the questionnaire across diverse professional settings, we cannot exclude self-selection bias, potentially resulting in the overrepresentation of practitioners with heightened interest in MIH. Furthermore, demographic distribution imbalances may exist within our sample. Although the questionnaire was piloted for clarity and estimated completion time, it did not undergo formal psychometric validation, which may impact the robustness of the instrument. These limitations warrant careful consideration when extrapolating our findings to the broader Romanian dental practitioner population. This study was limited to descriptive analysis; the lack of inferential statistical methods is acknowledged, and future research should explore deeper analytical approaches. Lastly, this study was geographically restricted to Romania, which, although it addresses a significant gap in national data on MIH, may limit the generalizability of the findings. The perceptions and clinical practices of Romanian dental professionals are likely shaped by specific local factors, including the structure of dental education, availability of continuing professional development, public health policies, and the national prevalence of MIH. As such, the results may not be directly applicable to international contexts with differing healthcare infrastructures or professional training frameworks.

## 5. Conclusions

This study offers critical insights into Romanian dental practitioners’ knowledge, clinical experience, and educational requirements regarding MIH.

MIH is a commonly encountered condition in dental practice, with 86.76% of surveyed dentists reporting clinical exposure to cases, indicating its significant prevalence and clinical relevance.

Despite the clinical significance of MIH, there exists a substantial educational gap, with only 35.16% of respondents having received formal training on the condition, contrasting sharply with the near-unanimous recognition (98.63%) of the need for additional education.

Our results emphasize the urgent need for structured educational programs focused on MIH diagnosis and management in both undergraduate curricula and continuing professional development.

Despite EAPD’s updated 2022 recommendations on MIH management, many practitioners may be either unaware of these guidelines or uninterested in implementing them.

Additionally, public awareness initiatives could promote earlier intervention, potentially improving long-term outcomes for affected patients.

Standardized clinical protocols specifically designed for MIH would benefit clinicians across specialties, particularly those involved in early diagnosis and treatment.

## Figures and Tables

**Table 1:** Distribution of respondents by dental specialization.

Specialization	Frequency (*n*)	Percentage (%)
General dentistry	117	53.42
Endodontics	43	19.63
Pediatric dentistry	25	11.42
Periodontology	18	8.22
Orthodontics	8	3.65
Oral surgery	5	2.28
Oral pathology	3	1.37
Total	219	100.00

**Table 2:** Perceived prevalence of MIH in the patient population.

Perceived Prevalence	Frequency (*n*)	Percentage (%)
High	63	33.16
Moderate	95	50.00
Low	32	16.84
Total	190	100.00

**Table 3:** Distribution of MIH-related complications by dental specialties.

Specialty	Pulp Exposure (%)	Hypersensitivity (%)	Failed Restorations (%)
General Dentists	35.35	37.37	27.27
Endodontists	38.71	48.39	12.90
Orthodontists	14.29	28.57	57.14
Periodontists	16.67	25.00	58.33
Oral surgeons	66.67	33.33	0.00
Pedodontists	21.05	73.68	5.26
Oral pathologists	66.67	33.33	0.00

**Table 4:** Preferred treatment approaches for MIH.

Treatment Option	Frequency (*n*)	Percentage (%)
Restorative treatments	133	70.00
Referral to specialist	21	11.05
Pulp therapy	17	8.95
Endodontic treatment	13	6.84
Other (e.g., prosthetics, fluoride therapy)	6	3.16
Total	190	100.00

**Table 5:** Preferred treatment approaches for MIH by most frequently reported dental specialties.

Specialty	Restorative Treatments (%)	Vital Pulp Therapy (%)	Endodontic Treatment (%)	Referral (%)
General Dentists	77.36	6.60	3.77	10.38
Endodontists	50.00	18.75	25.00	6.25
Orthodontists	57.14	0.00	0.00	42.86
Periodontists	69.23	0.00	7.69	15.38
Oral surgeons	50.00	0.00	0.00	50.00
Pedodontists	72.00	16.00	0.00	0.00
Oral pathologists	66.67	0.00	0.00	33.33

## Data Availability

The raw data supporting the conclusions of this article will be made available by the authors upon request.

## Abbreviations

The following abbreviations are used in this manuscript:

MIH	Molar Incisor Hypomineralization
HSPMs	Hypomineralized Secondary Primary Molars
EAPD	European Academy of Paediatric Dentistry
DMH	Deciduous Molar Hypomineralization
